# Oral Supplementation with *Z*-Isomer-Rich Astaxanthin Inhibits Ultraviolet Light-Induced Skin Damage in Guinea Pigs

**DOI:** 10.3390/md20070414

**Published:** 2022-06-24

**Authors:** Masaki Honda, Hakuto Kageyama, Yelin Zhang, Takashi Hibino, Motonobu Goto

**Affiliations:** 1 Faculty of Science & Technology, Meijo University, 1-501 Shiogamaguchi, Tempaku-ku, Nagoya 468-8502, Japan; kageyama@meijo-u.ac.jp (H.K.); ryelinz956@gmail.com (Y.Z.); hibino@meijo-u.ac.jp (T.H.); 2 Graduate School of Environmental and Human Sciences, Meijo University, Nagoya 468-8502, Japan; 3 Department of Materials Process Engineering, Nagoya University, Nagoya 464-8603, Japan; 4 Institute of Innovation for Future Society, Nagoya University, Furo-cho, Chikusa-ku, Nagoya 464-8601, Japan; goto.motonobu@material.nagoya-u.ac.jp

**Keywords:** Astaxanthin, geometrical isomer, UV-light irradiation, skin damage, elasticity, transepidermal water loss, melanin synthesis, guinea pigs

## Abstract

The effect of oral supplementation with astaxanthin of different *Z*-isomer ratios on ultraviolet (UV) light-induced skin damage in guinea pigs was investigated. Astaxanthin with a high *Z*-isomer content was prepared from the all-*E*-isomer via thermal isomerization. Intact (all-*E*)-astaxanthin and the prepared *Z*-isomer-rich astaxanthin were suspended in soybean oil and fed to guinea pigs for three weeks. The UV-light irradiation was applied to the dorsal skin on the seventh day after the start of the test diet supplementation, and skin parameters, such as elasticity, transepidermal water loss (TEWL), and pigmentation (melanin and erythema values), were evaluated. The accumulation of astaxanthin in the dorsal skin was almost the same after consumption of the all-*E*-isomer-rich astaxanthin diet (E-AST-D; total *Z*-isomer ratio = 3.2%) and the *Z*-isomer-rich astaxanthin diet (Z-AST-D; total *Z*-isomer ratio = 84.4%); however, the total *Z*-isomer ratio of astaxanthin in the skin was higher in the case of the Z-AST-D supplementation. Both diets inhibited UV light-induced skin-damaging effects, such as the reduction in elasticity and the increase in TEWL level. Between E-AST-D and Z-AST-D, Z-AST-D showed better skin-protective ability against UV-light exposure than E-AST-D, which might be because of the greater UV-light-shielding ability of astaxanthin *Z*-isomers than the all-*E*-isomer. Furthermore, supplementation with Z-AST-D resulted in a greater reduction in skin pigmentation caused by astaxanthin accumulation compared to that of E-AST-D. This study indicates that dietary astaxanthin accumulates in the skin and appears to prevent UV light-induced skin damage, and the *Z*-isomers are more potent oral sunscreen agents than the all-*E*-isomer.

## 1. Introduction

Astaxanthin (C_40_H_52_O_4_), which is found in various microorganisms (e.g., *Haematococcus pluvialis* and *Paracoccus carotinifaciens*) and seafoods (e.g., salmon and shrimp), is a dark-red pigment belonging to the xanthophyll subclass of carotenoids [1,2,3]. This carotenoid has potent antioxidant activity, 100-fold higher, for example, than that of α-tocopherol and 10-fold higher than that of other carotenoids, such as β-carotene and lutein [4,5]. Several studies have demonstrated that the daily intake of astaxanthin has excellent effects on human health and beauty [6,7,8,9,10]. In particular, astaxanthin has been reported to prevent ultraviolet (UV) light-induced skin damage and exhibits multiple biological activities that preserve skin health [7,8,9,10]. For example, Li et al. (2020) reported that astaxanthin supplementation reduced UV light-induced thickening and capillary regression in the skin of hairless mice [8]. Moreover, Ito et al. (2018) investigated the effects of dietary astaxanthin on UV light-induced skin deterioration in healthy Japanese participants and demonstrated that astaxanthin supplementation led to an increased minimal erythema dose and reduced the loss of skin moisture in the irradiated area compared with the placebo [9]. Thus, astaxanthin has a profound effect on skin health. However, the bioavailability of carotenoids, including astaxanthin, is generally very low [11,12]; therefore, to obtain maximum benefits, it is essential to improve their bioavailability after oral intake.

In recent years, several studies have reported that the “*Z*-isomerization” of carotenoids is very effective in improving their bioavailability (incidentally, most natural and synthetic carotenoids exist in the all-*E*-configuration) [12,13,14,15]. For example, an oral supplementation trial in rats fed with all-*E*-isomer-rich astaxanthin (total *Z*-isomer ratio = 0.5%) or *Z*-isomer-rich astaxanthin (total *Z*-isomer ratio = 80.8%) demonstrated that supplementation with *Z*-isomer-rich astaxanthin resulted in 36.8-and 12.2-fold increases in astaxanthin concentrations in the plasma and skin, respectively, compared to all-*E*-isomer-rich astaxanthin [15]. This study also showed that when rats were fed *Z*-isomer-rich astaxanthin, the total *Z*-isomer ratio of astaxanthin in the skin was significantly increased compared to that of rats fed all-*E*-isomer-rich astaxanthin; that is, the total *Z*-isomer ratios in the skin were approximately 50 and 10%, respectively [15]. Several studies have demonstrated that astaxanthin *Z*-isomers have greater antioxidant and anti-inflammatory activity levels than those of the all-*E*-isomer [16,17]. Therefore, there is a possibility that the intake of astaxanthin *Z*-isomers is preferable to that of the all-*E*-isomer for skin health. To our knowledge, no previous study has investigated the effects of oral supplementation with *Z*-isomer-rich astaxanthin on skin health and UV light-induced skin deterioration. As previous studies have used *H. pluvialis* as a source of astaxanthin [7,8,9,10], it is predicted that most astaxanthin was in the all-*E*-configuration [18]. This study aimed to evaluate the protective effects of astaxanthin with different *Z*-isomer ratios on UV light-induced skin deterioration. Specifically, the effects of oral supplementation with all-*E*-isomer-rich or *Z*-isomer-rich astaxanthin on UV light-induced changes in skin elasticity, transepidermal water loss (TEWL), and pigmentation were studied in guinea pigs. In addition, subjective skin conditions and epidermal thickness were analyzed using a visual analog scale.

## 2. Results

### 2.1. Profile of the Astaxanthin Diets

**Figure 1:**
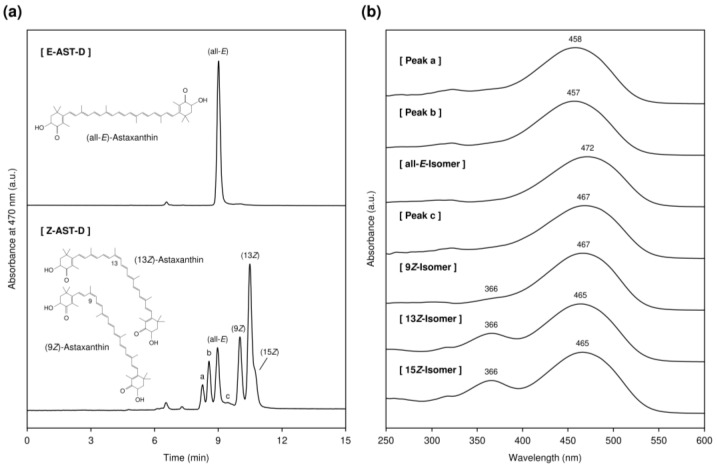
(**a**) Normal-phase HPLC chromatograms of an (all-*E*)-astaxanthin-rich diet (E-AST-D; total *Z*-isomer ratio = 3.2%) and a (*Z*)-astaxanthin-rich diet (Z-AST-D; total *Z*-isomer ratio = 84.4%) and (**b**) absorption spectra of peaks a–c and (all-*E*)-, (9*Z*)-, (13*Z*)-, and (15*Z*)-astaxanthin. Peaks a–c were tentatively identified as astaxanthin *Z*-isomers [19,20,21,22].

The HPLC chromatograms of an (all-*E*)-astaxanthin-rich diet (E-AST-D) and a (*Z*)-astaxanthin-rich diet (Z-AST-D) are shown in Figure 1a. The total *Z*-isomer ratios of E-AST-D and Z-AST-D were 3.2 and 84.4%, respectively. The predominant astaxanthin *Z*-isomers in Z-AST-D were the 9*Z*- and 13*Z*-isomers, representing 18.5% and 38.4%, respectively, of the total astaxanthin content. Moreover, the 15*Z*-isomer and three unidentified *Z*-isomers (possibly the di-*Z*-isomers [19,20,21,22]) were also present. The absorption spectra of astaxanthin isomers are shown in Figure 1b. In comparison with the spectrum of (all-*E*)-astaxanthin, the spectra of the *Z*-isomers shifted towards shorter wavelengths (Table S1). For the 9*Z*, 13*Z*-, and 15*Z*-isomers, in addition to the absorption spectrum characteristic of astaxanthin, a maximum absorption wavelength was observed around 366 nm, which is in the UV-A region (Figure 1b and Table S1).

### 2.2. Astaxanthin Concentration and Isomer Ratio in the Plasma and Dorsal Skin

**Figure 2:**
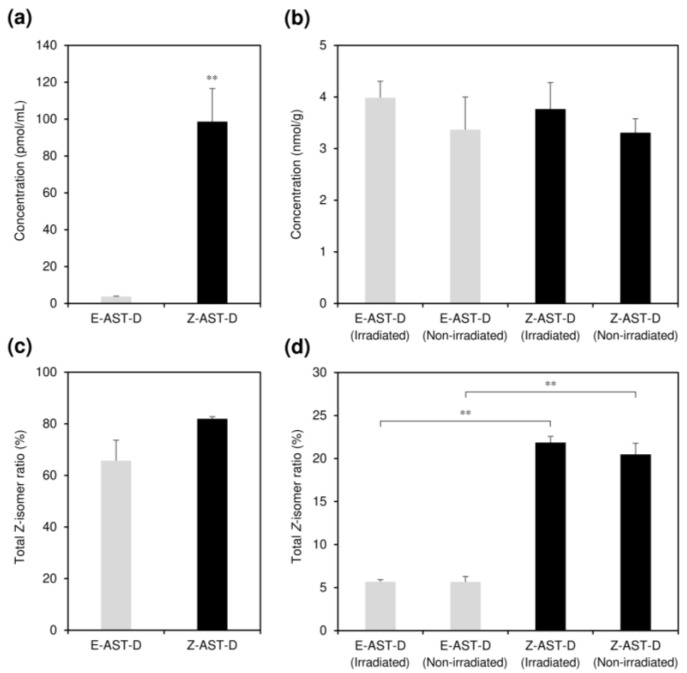
The effects of supplementation with E-AST-D and Z-AST-D on astaxanthin concentrations in (**a**) plasma and (**b**) dorsal skin with or without UV-light irradiation and total *Z*-isomers ratios of astaxanthin in (**c**) plasma and (**d**) dorsal skin with or without UV-light irradiation. Error bars represent the standard error of the mean (*n* = 3–9). Asterisks (*) indicate statistically significant differences in each group (** *p* < 0.01, Student’s *t*-test).

The effects of oral supplementation with astaxanthin of different *Z*-isomer ratios (E-AST-D and Z-AST-D) on astaxanthin concentration and isomer ratios in the plasma and dorsal skin of guinea pigs after the 13-day UV-light irradiation were evaluated (Figure 2 and Figure S1). Non-UV-irradiated areas of the dorsal skin were also examined. In the placebo group (supplemented with medium soybean oil), astaxanthin was not detected in the plasma and dorsal skin, whereas in those fed E-AST-D and Z-AST-D, astaxanthin isomers were detected. The supplementation with Z-AST-D resulted in 26.5 times higher plasma astaxanthin concentration than that of E-AST-D.

On the other hand, the astaxanthin concentration in the skin remained approximately constant regardless of the astaxanthin isomer ratio in the diet and whether it was with or without UV-light irradiation, e.g., the astaxanthin concentrations in the UV-irradiated skin after E-AST-D and Z-AST-D supplementation were 4.0 ± 0.3 and 3.7 ± 0.5 nmol/g, respectively. Astaxanthin in the plasma was rich in the *Z*-isomers, even after E-AST-D supplementation (total *Z*-isomer ratio = 65.7%). The total *Z*-isomer ratio of astaxanthin in the skin of guinea pigs fed with Z-AST-D was higher than that of guinea pigs fed with E-AST-D (approximately 20% and 5%, respectively). The total *Z*-isomer ratio of astaxanthin in the skin was lower than that in the plasma.

### 2.3. Effect of Astaxanthin Supplementation on Skin Elasticity

**Figure 3:**
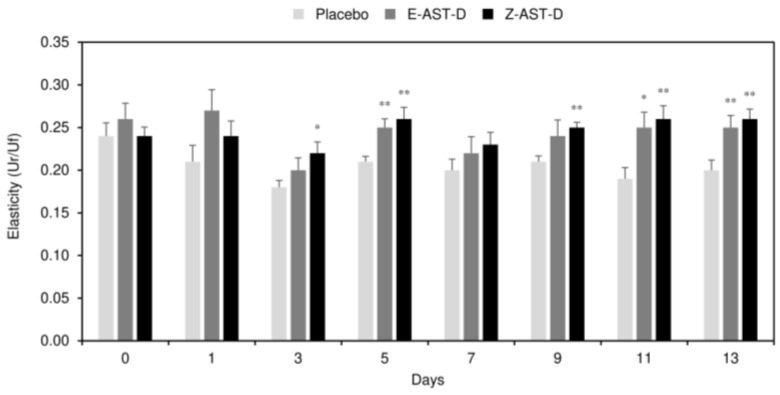
The effect of astaxanthin supplementation on the elasticity of UV-irradiated dorsal skin. Error bars represent the standard error of the mean (*n* = 9). Asterisks (*) indicate statistically significant differences from the placebo group (* *p* < 0.05 and ** *p* < 0.01, Student’s *t*-test).

The effect of astaxanthin supplementation on the elasticity of UV-irradiated dorsal skin is shown in Figure 3. The UV-light irradiation significantly decreased skin elasticity in the placebo diet group. Specifically, the elasticity value was 0.24 before irradiation, which decreased to ≤0.21 after the irradiation. In contrast, in the astaxanthin supplementation groups, the reduction in skin elasticity was significantly inhibited, and a stronger inhibitory effect was observed in the Z-AST-D group than in the E-AST-D group. In the E-AST-D group, the elasticity value was 0.26 before irradiation and decreased slightly to 0.25 after the 13-day irradiation, whereas in the Z-AST-D group, the elasticity value was 0.24 before the irradiation and increased to 0.26 after the 13-day irradiation.

### 2.4. Effect of Astaxanthin Supplementation on TEWL

**Figure 4:**
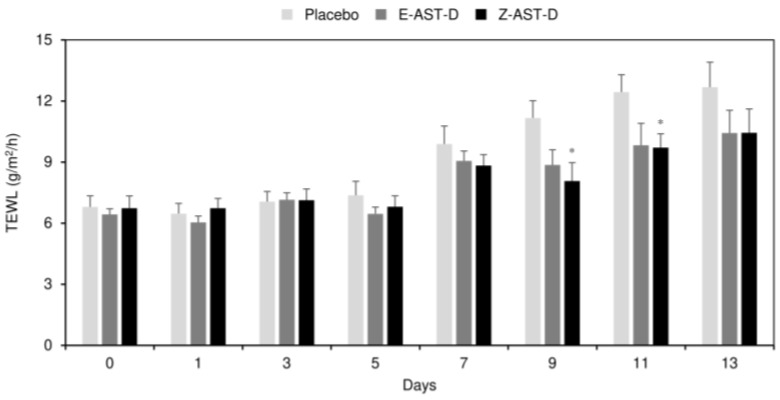
The effect of astaxanthin supplementation on the TEWL of UV-irradiated dorsal skin. Error bars represent the standard error of the mean (*n* = 9). Asterisks (*) indicate statistically significant differences from the placebo group (* *p* < 0.05, Student’s *t*-test).

The effect of dietary astaxanthin on the TEWL of UV-light-irradiated dorsal skin in guinea pigs is shown in Figure 4. An increase in the TEWL was observed from the seventh day after the start of irradiation therapy. Compared to the placebo, astaxanthin supplementation was more effective in preventing the increase in the TEWL. However, no significant difference in the TEWL was observed between the E-AST-D and Z-AST-D supplementation groups. For example, the TEWL values of the placebo, E-AST-D, and Z-AST-D groups after the 13-day irradiation were 12.7, 10.4, and 10.4 g/m^2^/h, respectively (the values before irradiation were 6.8, 6.4, and 6.7 g/m^2^/h, respectively).

### 2.5. Effect of Astaxanthin Supplementation on Skin Pigmentation

**Figure 5:**
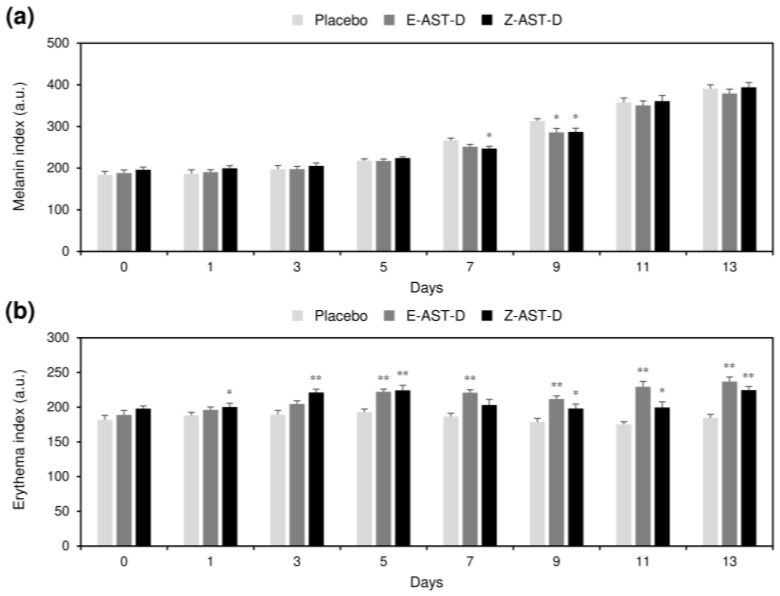
The effect of astaxanthin supplementation on the pigmentation of UV-irradiated dorsal skin. (**a**) Melanin and (**b**) erythema values were measured as the skin pigmentation indicator. Error bars represent the standard error of the mean (*n* = 9). Asterisks (*) indicate statistically significant differences from the placebo group (* *p* < 0.05 and ** *p* < 0.01, Student’s *t*-test).

**Figure 6:**
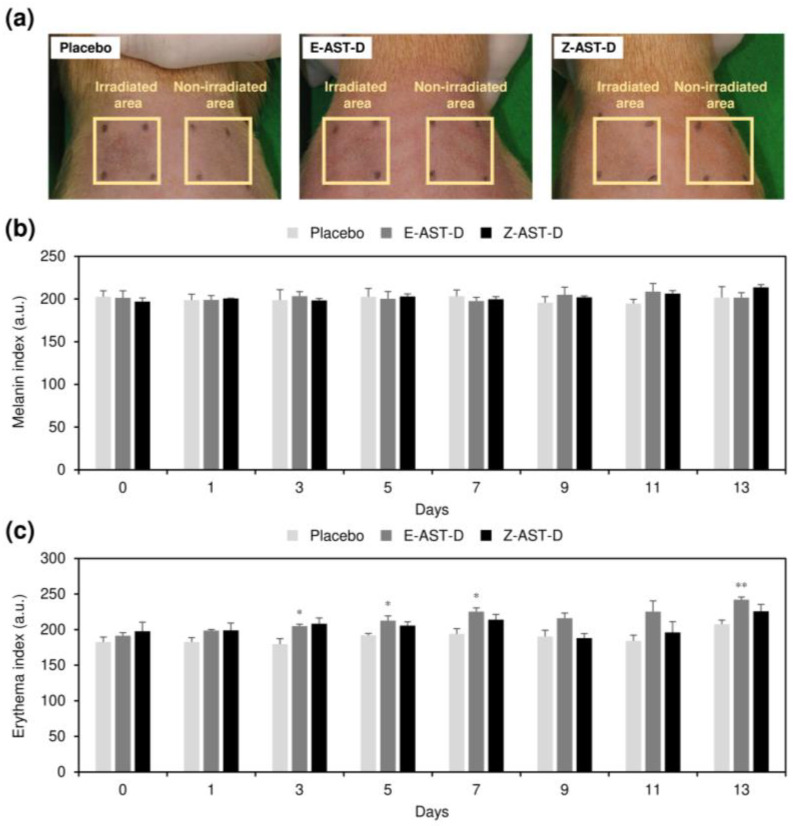
(**a**) Typical photographs of the 7-day UV-irradiated dorsal skin with UV-irradiated and non-UV-irradiated areas and changes in (**b**) melanin and (**c**) erythema values of non-UV-irradiated dorsal skin area. Error bars represent the standard error of the mean (*n* = 3). Asterisks (*) indicate statistically significant differences from the placebo group (*p < 0.05 and **p < 0.01, Student’s *t*-test).

The effect of oral supplementation with E-AST-D and Z-AST-D on the pigmentation of UV-irradiated dorsal skin was investigated. The skin pigmentation was evaluated based on melanin and erythema values (Figure 5). The melanin value increased with the UV-light exposure, and when E-AST-D and Z-AST-D were administered, the increases in melanin values at the seventh and ninth days after the start of the irradiation were slightly inhibited (Figure 5a). No significant difference in melanin inhibition was observed between the E-AST-D and Z-AST-D groups. Incidentally, the melanin value did not increase during the test period in the skin area that was not exposed to UV light (Figure 6a,b). Supplementation with E-AST-D and Z-AST-D resulted in an increase in the erythema value (Figure 5b). This increase was significant from the third day after the start of UV-light irradiation, and the degree of increase was greater in the E-AST-D group than in the Z-AST-D group. Erythema values did not increase in the placebo group. When E-AST-D and Z-AST-D were administered, the erythema value also increased in the skin area that was not irradiated with the UV light, and the degree of increase was higher in the E-AST-D group (Figure 6a,c).

### 2.6. Effect of Astaxanthin Supplementation on Skin Conditions

**Figure 7:**
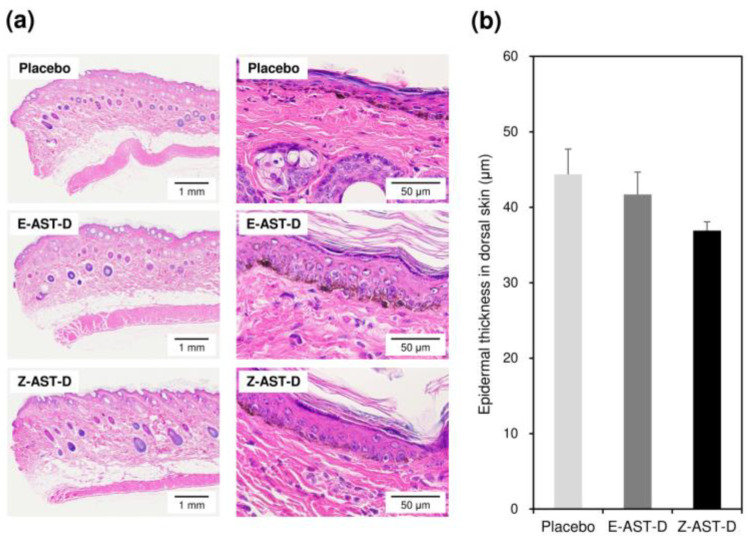
(**a**) Typical photographs of the 13-day UV-irradiated dorsal skin stained with hematoxylin and eosin and (**b**) epidermal thickness of the dorsal skin after the 13-day irradiation therapy (Figure S2). Error bars represent the standard error of the mean (*n* = 3).

Subjective skin conditions were assessed on the 13-day UV-irradiated dorsal skin samples stained with hematoxylin and eosin (Figure 7a). In the placebo group, increases in melanin granules and sunburn cells and atrophy were observed in the epidermal layer, while inflammatory cell infiltration and hemorrhage were observed in the dermal layer. In the E-AST-D group, the aforementioned skin conditions were almost similar to those in the placebo group, whereas in the Z-AST-D group, sunburn cells and hemorrhage were not observed. Astaxanthin supplementation showed an inhibitory effect on epidermal thickening caused by UV-light exposure, and this effect was stronger in the Z-AST-D supplementation group than in the E-AST-D supplementation group—that is, the average epidermal thicknesses of UV-irradiated dorsal skin samples in the placebo, E-AST-D, and Z-AST-D groups were 44.4, 41.7, and 36.9 μm, respectively (Figure 7b).

## 3. Discussion

Supplementation with Z-AST-D led to a higher increase in the blood astaxanthin levels of guinea pigs compared to supplementation with E-AST-D. Thus, the astaxanthin *Z*-isomers demonstrated higher bioavailabilty than the all-*E*-isomer, similar to that observed in our recent study using rats and hens [15,23]. In contrast, the astaxanthin level in the dorsal skin remained approximately constant regardless of the astaxanthin isomer ratio. This result differs from that of our previous study using rats [15]. When rats were fed *Z*-isomer-rich astaxanthin, the astaxanthin concentration in the skin was 12.2 times higher than that of rats fed all-*E*-isomer-rich astaxanthin. The difference in astaxanthin accumulation in the skin between guinea pigs and rats may be due to differences in animal species. Several studies have demonstrated that the absorption characteristics of astaxanthin isomers in living bodies differ among species (e.g., rats, hens, salmon, and halibut) [15,23,24].

Alternatively, the dose of astaxanthin administered in this study (10 mg of astaxanthin/kg body weight) was so high that the accumulation in the guinea pig skin may have reached the upper limit. Future studies with lower doses will be needed to prove this. On the other hand, when guinea pigs were fed Z-AST-D, the total *Z*-isomer ratio in the skin was higher than that with E-AST-D supplementation. However, the ratio in the skin was significantly lower compared to that introduced through the diet; that is, the total *Z*-isomer ratios of Z-AST-D and skin astaxanthin were 84.4 and 21.9%, respectively. This trend has also been confirmed in tests using rats, suggesting that there is a mechanism for maintaining a certain ratio of carotenoid *Z*-isomers in the body [15,25].

**Figure 8:**
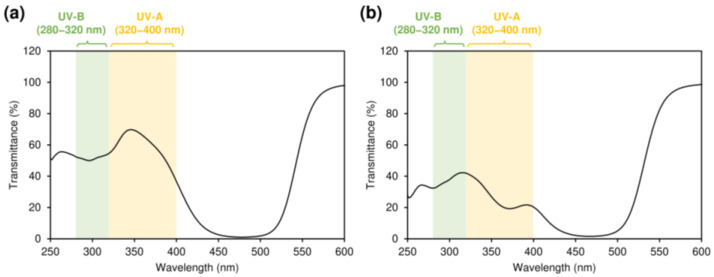
Transmittance of (**a**) all-*E*-isomer-rich astaxanthin and (**b**) *Z*-isomer-rich astaxanthin. The astaxanthin samples were dissolved in ethyl acetate at a concentration of 0.01 mg/mL.

Supplementation with E-AST-D and Z-AST-D inhibited the reduction in elasticity and the increase in the TEWL by UV-light irradiation. It also tended to inhibit melanin synthesis and skin thickening. Although many studies have reported similar results regarding the protective effects of astaxanthin supplementation on the skin, they used *H. pluvialis*-derived astaxanthin (mainly the all-*E*-isomer) [7,8,9,10,18]. In this study, we evaluated the protective effect of *Z*-isomer-rich astaxanthin against UV light-induced skin damage for the first time and observed that the *Z*-isomers have a notable effect on that, as with the all-*E*-isomer. The results of the skin elasticity evaluation and the tissue examinations, including epidermal thicknesses, indicate that astaxanthin *Z*-isomers potentially have a greater skin-protective effect from UV-light exposure than the all-*E*-isomer. When guinea pigs were fed E-AST-D and Z-AST-D, the concentration of astaxanthin accumulated in the skin was almost the same (Figure 2b), but the total *Z*-isomer ratio of astaxanthin in the skin was higher in the case of Z-AST-D supplementation (Figure 2d). Astaxanthin *Z*-isomers have shorter wavelengths than the all-*E*-isomer and have a characteristic maximum absorption spectrum at 366 nm (Figure 1b). Thus, it is considered that astaxanthin *Z*-isomers absorb UV light more efficiently, which might effectively inhibit UV light-induced skin damage. In fact, we confirmed that the UV-light-shielding ability of *Z*-isomer-rich astaxanthin (total *Z*-isomer ratio = 89.8%) was higher than that of all-*E*-isomer-rich astaxanthin (total *Z*-isomer ratio = 1.8%), that is, the UV-A shielding rates of all-*E*- and *Z*-isomer-rich astaxanthin (0.01 mg/mL in ethyl acetate) were 40.5 and 73.4%, respectively, and their UV-B shielding rates were 48.2 and 60.6%, respectively (Figure 8). Moreover, several studies have demonstrated that astaxanthin *Z*-isomers exhibit more potent antioxidant and anti-inflammatory activities than the all-*E*-isomer [16,17], which might have contributed to the superior skin-protective effect of Z-AST-D.

Astaxanthin supplementation induced an increase in the erythema value of the UV-irradiated dorsal skin (Figure 5b), but no increase was observed in the placebo group. Moreover, the erythema value increased in the skin area that was not irradiated with UV light (Figure 6c). Thus, it is considered that the increase in erythema values was not due to UV irradiation, but due to the deposition of astaxanthin in the skin. Ample studies have demonstrated that long-term intake of high concentrations of carotenoids results in skin pigmentation [26,27]. Interestingly, the erythema value was higher with E-AST-D supplementation than with Z-AST-D supplementation, although the astaxanthin concentration in the skin was almost the same (Figure 2b). This might be because carotenoid *Z*-isomers have lower molar absorption coefficients than the all-*E*-isomers and have shorter wavelengths of the absorption spectra compared to that of the all-*E*-isomers [19,20,28], that is, the chromogenic property of the *Z*-isomers is lower than that of the all-*E*-isomers. In addition, we investigated color tones (i.e., *L*, *a*, and *b* values) of all-*E*-isomer- and *Z*-isomer-rich astaxanthin for further discussion of the differences in skin pigmentation between them. The *L*, *a*, and *b* values of all-*E*-isomer-rich astaxanthin (0.01 mg/mL in ethyl acetate) were 77.7, 29.7, and 48.2, respectively, and those of *Z*-isomer-rich astaxanthin (0.01 mg/mL in ethyl acetate) were 83.3, 15.1, and 52.7, respectively. The higher *a* value of all-*E*-isomer-rich astaxanthin indicates higher redness and greenness than the *Z*-isomer-rich astaxanthin. Hence, the oral intake of Z-AST-D for skincare purposes is superior to E-AST-D intake in the following two aspects: inhibition of UV light-induced skin damage and reduction of skin pigmentation caused by the accumulation of astaxanthin. Furthermore, consumption of Z-AST-D increases the blood astaxanthin concentration more than consumption of E-AST-D, which might result in a variety of health benefits, such as a reduced risk of cancer and chronic diseases [29,30]. As mentioned above, the accumulation efficiency of astaxanthin *Z*-isomers in the skin of rats is higher than that in guinea pigs. Thus, it is possible that the skin-protective effect of Z-AST-D against UV light-induced skin damage is more pronounced in rats. It is expected that the bioavailability and skin accumulation characteristics of astaxanthin isomers will be determined in various animals, including humans, and that their effects on UV light-induced skin damage will be evaluated.

## 4. Materials and Methods

### 4.1. Materials

High-purity (all-*E*)-astaxanthin standard, high-performance liquid chromatography (HPLC)-grade organic solvents (hexane, ethyl acetate, acetone, and dichloromethane [CH_2_Cl_2_]), and soybean oil were purchased from FUJIFILM Wako Pure Chemical Corp. (Osaka, Japan). Analytical-grade ethanol and butylated hydroxytoluene (BHT) were purchased from Kanto Chemical Co., Inc. (Tokyo, Japan).

### 4.2. Diet Preparation

Astaxanthin rich in the *Z*-isomers was prepared from the all-*E*-isomer standard via thermal treatment and solid-liquid separation, as described in our previous study [14,15]. Briefly, a 2 mg/mL (all-*E*)-astaxanthin solution in CH_2_Cl_2_ was heated at 80 °C for 4 h for the *Z*-isomerization, and the solution was evaporated to dryness using a rotary evaporator (EYELA N-1300, Tokyo Rikakikai Co., Ltd., Tokyo, Japan) under reduced pressure at 40 °C. The resulting solid was suspended in ethanol and stored at 4 °C for 1 h to crystallize and precipitate the all-*E*-isomer. The insoluble substance, mainly composed of the all-*E*-isomer, was removed using a 0.22-μm PTFE filter (Osaka Chemical Co., Ltd., Osaka, Japan). After removing the ethanol from the filtrate using an evaporator under reduced pressure at 40 °C, *Z*-isomer-rich astaxanthin was obtained. Intact (all-*E*)-astaxanthin and prepared *Z*-isomer-rich astaxanthin were suspended in soybean oil at a concentration of 10 mg/mL using an ultrasonic homogenizer (UP-200P; Tomy Seiko Co., Ltd., Tokyo, Japan). These soybean-oil-based E-AST-D and Z-AST-D were used in the animal tests.

### 4.3. Animals and Treatments

Animal experiments were performed at I Tech Lab. Co., Ltd. (Gifu, Japan). The protocols for animal experiments were approved by the Institutional Animal Care and Use Committee of the I Tech Lab (Permission No. ITL-21-GO-319). Female Weiser-Maples guinea pigs (8-week-old) were purchased from Japan SLC Inc. (Shizuoka, Japan). The guinea pigs were housed in an animal room at a temperature of 23 ± 5 °C and humidity of 55 ± 25% with a 12-h light-dark cycle. Animals were allowed free access to an astaxanthin-free standard laboratory diet (0525; Japan SLC Inc., Shizuoka, Japan) and water. After 1 week of acclimation with the standard diet, the guinea pigs were randomly assigned to three groups: placebo (soybean oil), E-AST-D (10 mg of all-*E*-isomer-rich astaxanthin/kg body weight), and Z-AST-D (10 mg of *Z*-isomer-rich astaxanthin/kg body weight). They were fed the diets via intragastric intubation daily for 3 weeks. The UV irradiation was performed using a UV lamp (Dermaray-200 type equipped with UV-A and UV-B lamps; Terumo Clinical Supply Co., Ltd., Gifu, Japan) and the distance from the lamp to the guinea pigs was adjusted so that the average UV-B light intensity was 0.8 mW/cm^2^ [31,32,33]. The guinea pigs were shaved on their backs and irradiated with a UV lamp (2 cm × 2 cm), and the non-irradiated area was covered with aluminum foil. During irradiation, living guinea pigs were immobilized so that they could not move. The UV irradiation was applied for 8 min per day on the 8th, 10th, 12th, 14th, 16th, and 18th days after the initiation of test diet supplementation. Guinea pigs were sacrificed after three days from the last UV irradiation (i.e., the 21st day after the initiation of test diet supplementation) by exsanguination under anesthesia, and blood and dorsal skin samples were collected. Plasma samples were obtained from heparin-treated blood by centrifugation at 1700*× g* for 15 min [14,15]. The collected plasma and skin samples were stored at −80 °C until analysis.

### 4.4. Evaluation of Dorsal Skin

The dorsal skin condition was evaluated on the 8th, 9th, 11th, 13th, 15th, 17th, 19th, and 21st days after the initiation of test diet supplementation, that is, immediately before the UV-light irradiation and on the 1st, 3rd, 5th, 7th, 9th, 11th, and 13th days after the initiation of UV irradiation. Skin elasticity was measured using a Cutometer (MPA 580; Integral Corp., Tokyo, Japan), TEWL was analyzed using a Tewameter (TM300; Integral Corp., Tokyo, Japan), and skin pigmentation (melanin and erythema values) was assessed using a Mexameter (MX18; Integral Corp., Tokyo, Japan) [34,35]. Subjective skin conditions were assessed by experts from Applied Medical Research (Osaka, Japan) on 13-day UV-irradiated dorsal skin samples stained with hematoxylin and eosin. The epidermal thickness of the dorsal skin was measured using an image analysis system (WinROOF2018; Mitani Co., Ltd., Tokyo, Japan). The epidermal layer was defined as the stratum granulosum, stratum spinosum, and stratum basale, excluding the stratum corneum (Figure S2).

### 4.5. Extraction of Astaxanthin Isomers from the Plasma and Skin

Extraction of astaxanthin isomers from plasma and skin samples was performed according to a previously described method [14,15]. Astaxanthin isomers were extracted from the plasma samples using hexane containing 0.01% BHT with a vortex mixer. Prior to extraction, the same volume of ethanol as the collected plasma was added to precipitate the proteins. Hexane and water were separated with centrifugal separation treatment and the hexane layer containing astaxanthin isomers was carefully collected. This hexane extraction was carried out three times for each plasma sample at room temperature and then hexane was removed using a nitrogen gas stream. The resulting solid was dissolved in hexane/ethyl acetate (70:30, *v/v*) and filtered through a 0.22-μm PTFE membrane filter before HPLC analysis. Astaxanthin isomers in skin samples were extracted using acetone containing 0.01% BHT. Extraction was carried out via ultrasonication (CPX1800H-J; Yamato Scientific Co., Ltd., Tokyo, Japan) for 15 min in an ice water bath (approximately 4 °C) to prevent the thermal isomerization of astaxanthin isomers. The extracted liquid was filtered through a 0.22-μm PTFE membrane filter, and the resulting filtrate was evaporated to dryness using a rotary evaporator. The resulting substance was dissolved in hexane/ethyl acetate (70:30, *v/v*), filtered through a 0.22-μm PTFE membrane filter, and then analyzed by HPLC.

### 4.6. Analysis of Astaxanthin Isomers

Astaxanthin isomers in the plasma and skin of guinea pigs were analyzed by normal-phase HPLC equipped with two silica gel columns connected in tandem (Luna 5 μm Silica (2), 2 × 150 mm × 4.6 mm, 100 Å, Phenomenex Inc., Torrance, CA, USA) as previously described [15,19]. Briefly, a mixture of hexane/ethyl acetate/acetone (70:20:10, *v/v/v*) was used as the mobile phase, and the flow rate was maintained at 1.2 mL/min. Astaxanthin isomers were detected using a photodiode array detector (SPD-M20A, Shimadzu Corp., Kyoto, Japan) at 470 nm and peak identification was performed based on HPLC retention times, spectral data, and relative intensities of the *Z*-peak to the absorption maximum peak of the isomer (*Q*-ratio) (Table S1) [19,20,21,22]. The UV-light-shielding abilities and color indices (Hunter *L* [lightness], *a* [redness and greenness], and *b* [yellowness and blueness]) of all-*E*-isomer-rich astaxanthin (total *Z*-isomer ratio = 1.8%) and *Z*-isomer-rich astaxanthin (total *Z*-isomer ratio = 89.8%) were assessed using a UV-vis spectrophotometer (V-750, Jasco Corp., Tokyo, Japan) equipped with UV shield factor calculation and color calculation programs. For this, the astaxanthin samples were dissolved in ethyl acetate at a concentration of 0.01 mg/mL. The UV-light-shielding ability was assessed in UV-A (320–400 nm) and UV-B (280–320 nm) regions (Figure 8).

### 4.7. Statistical Analysis

Data are reported as mean ± standard error of the mean of more than three experimental skin samples. The differences in mean values were analyzed by Student’s *t*-test with the EZR software program (version 1.54; Saitama Medical Center, Jichi Medical University, Saitama, Japan), and significant differences were calculated at *p* < 0.05 or *p* < 0.01.

## 5. Conclusions

When guinea pigs were fed E-AST-D and Z-AST-D, the accumulation of astaxanthin in the dorsal skin was almost the same between the two dietary groups; however, the total *Z*-isomer ratio of astaxanthin in the skin was higher in the case of Z-AST-D supplementation. Astaxanthin supplementation significantly suppressed UV light-induced skin-damaging effects, such as reduced elasticity and increased TEWL levels. A comparison between E-AST-D and Z-AST-D indicated that Z-AST-D was more effective in suppressing UV light-induced skin damage. This may be because astaxanthin *Z*-isomers have a higher UV light-shielding ability than the all-*E*-isomer. Moreover, Z-AST-D supplementation resulted in a greater reduction in skin pigmentation caused by astaxanthin accumulation compared with E-AST-D supplementation. Hence, the intake of *Z*-isomer-rich astaxanthin is considered very effective in suppressing UV light-induced skin damage. To ensure the usefulness of astaxanthin *Z*-isomers for skin health, it is necessary to conduct human and other animal trials in the future.

## Data Availability

Not applicable.

## Supplementary Materials

The following supporting information can be downloaded at: https://www.mdpi.com/article/10.3390/md20070414/s1, Figure S1: Normal-phase HPLC chromatograms of astaxanthin isomers in plasma and dorsal skin of the guinea pigs; Figure S2: Photographs of dorsal skin stained with hematoxylin and eosin showing the epidermal area and the image of the extracted epidermal area; Table S1: Absorption maxima and relative intensities of the *Z*-peaks (*Q*-ratio) of astaxanthin isomers separated and observed using normal-phase HPLC.
