# Supplementary material for: Oral Supplementation with Z-Isomer-Rich Astaxanthin Inhibits Ultraviolet Light-Induced Skin Damage in Guinea Pigs
Source: Mar Drugs. 2022 Jun 24;20(7):414. doi: 10.3390/md20070414 (PMC9315510; doi:10.3390/md20070414)
Supplement: Supplementary file 1 [file marinedrugs-20-00414-s001.zip › marinedrugs-1776345-supplementary.pdf]

Supporting information

Oral Supplementation with *Z*-Isomer-Rich Astaxanthin Inhibits Ultraviolet Light-Induced Skin Damage in Guinea Pigs

Masaki Honda [1,*], Hakuto Kageyama [1,2], Yelin Zhang [1,3], Takashi Hibino [1,2], and Motonobu Goto [4]

[1] Faculty of Science & Technology, Meijo University, 1-501 Shiogamaguchi, Tempaku-ku, Nagoya 468-8502, Japan

[2] Graduate School of Environmental and Human Sciences, Meijo University, Nagoya 468-8502, Japan

[3] Department of Materials Process Engineering, Nagoya University, Nagoya 464-8603, Japan

[4] Institute of Innovation for Future Society, Nagoya University, Furo-cho, Chikusa-ku, Nagoya 464-8601, Japan

* Correspondence: honda@meijo-u.ac.jp; Tel.: +81-52-838-2284

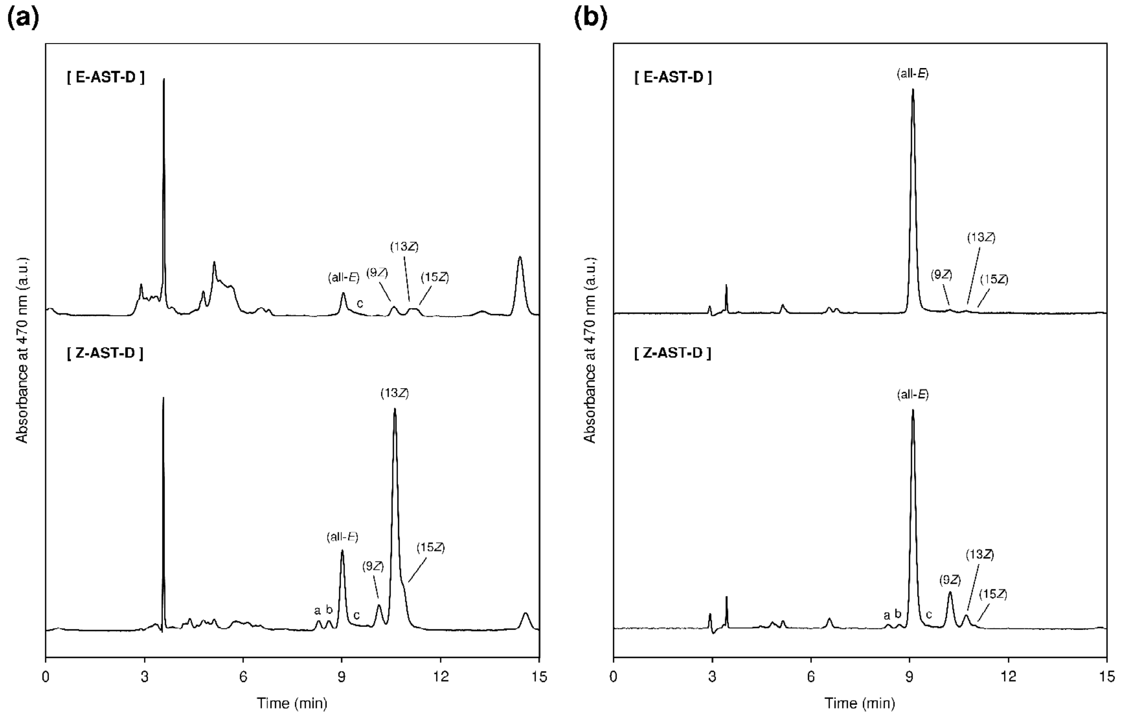

Figure S1. Normal-phase HPLC chromatograms of astaxanthin isomers in (**a**) plasma and (**b**) dorsal skin of the guinea pigs after the feeding of (all-*E*)-astaxanthin-rich diet (E-AST-D) and (*Z*)-astaxanthin-rich diet (Z-AST-D) with UV light irradiation. Peaks a–c were tentatively identified as astaxanthin *Z*-isomers [19–22].

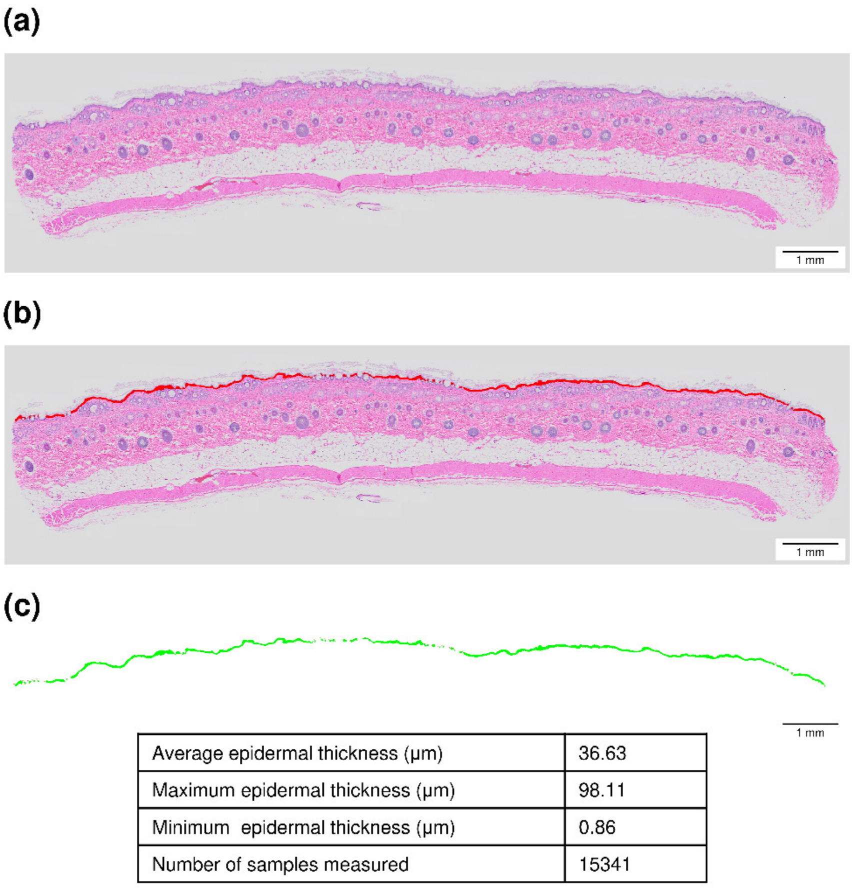

Average epidermal thickness (μm)	36.63
Maximum epidermal thickness (μm)	98.11
Minimum epidermal thickness (μm)	0.86
Number of samples measured	15341

Figure S2. Photographs of dorsal skin stained with hematoxylin and eosin (**a**) before and (**b**) after showing the epidermal area, and (**c**) the image of the extracted epidermal area used for the measurement.

Table S1

Absorption maxima (λ_{max}) and relative intensities of the *Z*-peaks (*Q*-ratio) of astaxanthin isomers separated and observed using normal-phase high-performance liquid chromatography (HPLC)[1]

		λ_{max} (nm)		*Q*-ratio	
Peaks	Isomers[2]	Observed	Reported[2]	Observed	Reported[2]
a	Unidentified *Z*-isomer	458	458	ND	ND
b	Unidentified *Z*-isomer	457	458	ND	ND
(all-*E*)	all-*E*-Isomer	472	472	ND	ND
c	Unidentified *Z*-isomer	466	457	ND	ND
(9*Z*)	9*Z*-Isomer	366, 467	366, 465	0.21	0.21
(13*Z*)	13*Z*-Isomer	366, 464	366, 464	0.49	0.47
(15*Z*)	15*Z*-Isomer	366, 466	365, 464	0.54	0.54

[1] Values and peak designations were obtained from the chromatograms in Figure 1a. ND, Not detected substantially.

[2] Tentatively assigned in the literature [19–22].
